# Genetic variations in LIGHT are associated with susceptibility to ankylosing spondylitis in a Chinese Han population

**DOI:** 10.18632/oncotarget.20644

**Published:** 2017-09-05

**Authors:** Bin Yang, Junlong Zhang, Lixin Li, Xiaojun Lyu, Wei Wei, Zhuochun Huang, Bei Cai, Lanlan Wang

**Affiliations:** ^1^ Department of Laboratory Medicine, West China Hospital, Sichuan University, Sichuan 610041, China; ^2^ West China School of Medicine, Sichuan University, Sichuan 610041, China

**Keywords:** genetic polymorphisms, LIGHT, BTLA, ankylosing spondylitis

## Abstract

Ankylosing spondylitis (AS) is a common chronic autoimmune disease characterized by inflammation of axial skeleton and has strong genetic susceptibility. Single nucleotide polymorphisms (SNPs) have been found playing an important role in the development of AS. This study intends to explore whether the susceptibility to AS is associated with rs2171513 C>T, rs1077667 G>A in LIGHT (lymphotoxin, expressed on T lymphocytes) and rs12609318 A>G in B and T lymphocyte attenuator (BTLA) in a Chinese Han population. We studied a total of 497 AS patients and 387 healthy controls in the current research. Clinical characteristics were recorded when they were recruited. Single nucleotide polymorphisms (SNPs) were genotyped by polymerase chain reaction (PCR) and high-resolution melting methods (HRM). Statistically significant difference was found in both co-dominant model (GG vs. GA vs. AA) (p = 4.00E-06) and alleles (p = 4.59E-08) of rs1077667 between patients and controls. There was also a significant difference in alleles of rs2171513 (p = 0.037) between patients and controls. We found rs1077667 in LIGHT and rs2171513 in BTLA with susceptibility to AS, while 12609318 in LIGHT associate with susceptibility to AS. Our results showed that LIGHT might be involved in pathogenesis of AS.

## INTRODUCTION

Ankylosing spondylitis (AS) is a common chronic autoimmune disease characterized by axial skeleton arthritis and enthesitis [1]. Axial skeleton impairment and hypofunction decrease social and working ability of patients and make them suffering. Previous studies have shown that single nucleotide polymorphisms (SNPs) associated with the development of AS. Identification of genes underlying AS is vital to our understanding of its etiology and helpful for prophylaxis, diagnosis and treatment of AS [2, 3].

Disarrangement of immune homeostasis can induce development of autoimmune disease. Cytokines and their pathways are important for maintaining immune homeiostasis [4]. Tumor necrosis factor receptor superfamily (TNFRSF) member herpes virus entry mediator (HVEM, also known as TNFRSF14) is a very special cytokine [5]. HVEM regulates immune response of T-cell in both stimulatory and inhibitory signaling pathways by combining with different ligands. Experimental mouse models and human diseases have indicated the correlation between dysregulation of HVEM network and autoimmune pathogenesis. HVEM is now considered as an attractive target for drug intervention. LIGHT(lymphotoxin exhibits inducible expression and competes with herpes simplex virus glycoprotein D for HVEM, a receptor expressed on T lymphocytes)acts as a ligand and binds to HVEM to stimulate inflammation [6, 7], whereas the engagement between HVEM and B and T lymphocyte attenuator (BTLA), a member of the immunoglobulin superfamily, mediates inhibitory signaling pathway [8].

As long as the exact mechanism of the genetic susceptibility to AS has not been fully discovered, development of AS will not be completely understood. SNP, a bi-allelic type of marker used in genetic studies with autofrequency, has been proved its efficacy in testifying the genetic susceptibility to diseases in recent years [9–11]. We chose three SNPs in LIGHT and BTLA to figure out whether they have any impact on the susceptibility to AS.

Above all, our study aimed to investigate the correlation between AS and three SNPs, rs2171513 C>T in BTLA, rs1077667 G>A and rs12609318 A>G in LIGHT in a Chinese Han population.

## RESULTS

### Demographic data

Population stratification and sampling bias in patients and controls were not in HWE (*p*< 0.05), but matches up with the data shown in HapMap. The mean ages of patients and controls were 33.1 ± 11.3 (range 10 to 74) years old and 37.8 ± 14.3 (range 13 to 77) years old, respectively. Males account for 69% (342/497) of patients and females 31% (155/497) for patients and 69% (266/387) are male and 31% (121/387) are female for controls. Age and gender does not have any significant difference between patients and controls (*p* = 0.811 and 0.979931 respectively) (Table 1) [12].

**Table 1:** Clinical characteristics of patients and controls

Characteristics	Healthy controls	AS patients	p
Gender (male : female)	266 :121	342 : 155	0.979931
Age (years)	37.84 ± 14.31	33.10 ± 11.27	0.811
CRP (mg/L)	---	20.66(2.89, 24.60)	
WBC (10^9^/L)	---	7.66 ± 2.00	
RBC (10^12^/L)	---	4.92 ± 0.60	
PLT (10^9^/L)	---	252 ± 95	
C3(g/L)	---	1.10 ± 0.28	
C4(g/L)	---	0.25 ± 0.09	
MONO# (10^9^/L)	---	0.43 ± 0.16	
LYMPH#(10^9^/L)	---	2.00 ± 0.61	
NEUT#(10^9^/L)	---	4.98 ± 1.77	

CRP: C-reactive protein, WBC: white blood cell, RBC: red blood cell, PLT: platelet, MONO#: monocyte, LYMPH#: lymphocyte, NEUT#: neutrophil, C3: complement 3, C4: complement 4.

The data are presented as mean ± SD.

p< 0.05 is considered of statistical significance.

### Determination of rs2171513, rs1077667 and rs12609318 genotypes

**Figure 1:**
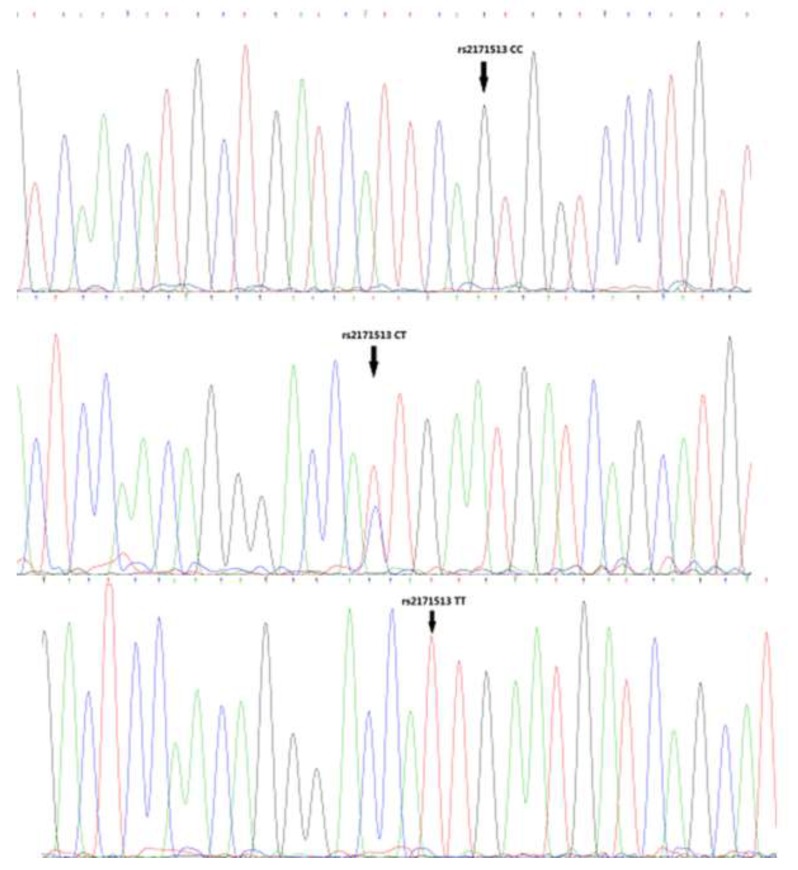
Representative sequencing results of rs2171513

**Figure 2:**
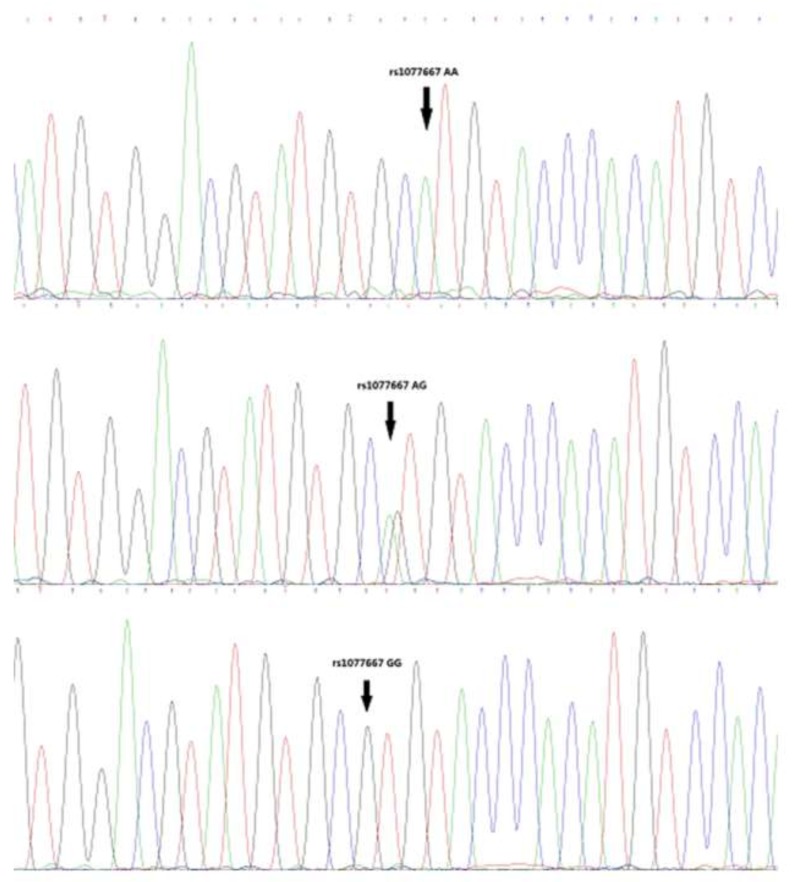
Representative sequencing results of rs1077667

**Figure 3:**
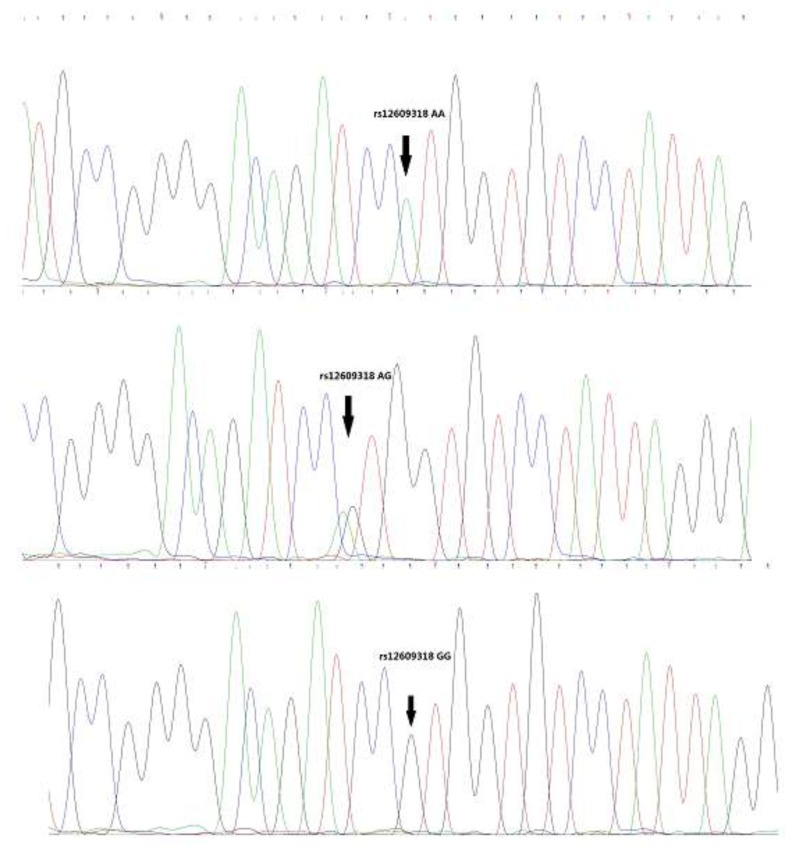
Representative sequencing results of rs12609318

**Figure 4:**
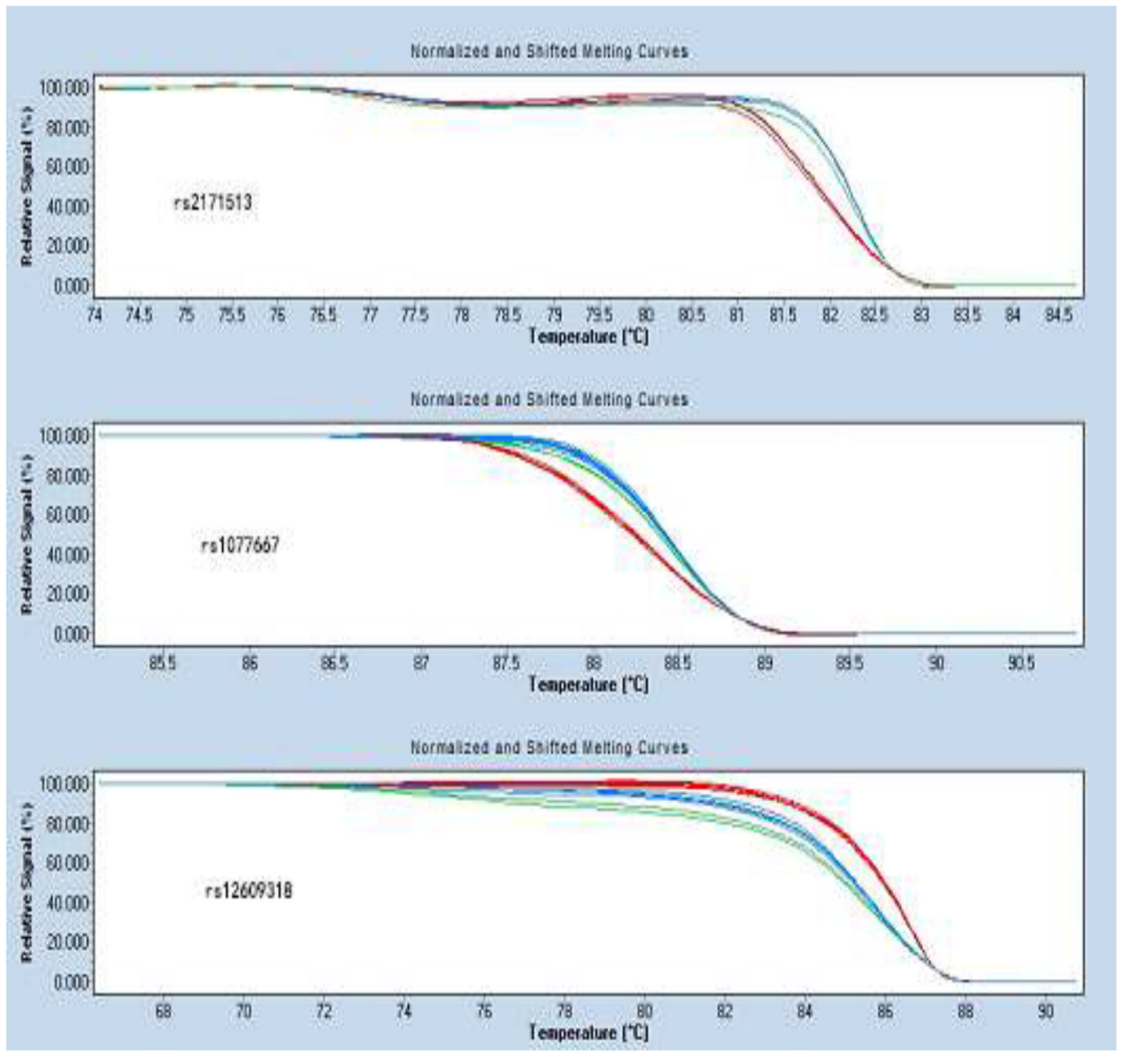
Normalized and shifted melting curves of rs2171513, rs1077667 and rs12609318

Representative sequencing results are shown in Figures 1-3. Three genotype melting profiles of each SNP (rs2171513, rs1077667 and rs12609318) were clearly distinguished from the normalized melting curves. Additional PCR products were randomly selected from each of the three genotype melting profiles (Figure 4).

### rs1077667 has association with the susceptibility to AS in both genotype and allele

Association was found between genotypes of rs1077667 and susceptibility to AS(p=4.00E-06, power = 98%). The frequency of AA genotype is significantly lower in controls than that in AS patients [OR (95% CI) = 0.578 (0.460-0.726)]. As for alleles, the frequency of A allele ofrs1077667 is lower in the controls (controls vs. patients 37.1% vs. 50.1%, *p* =4.59E-08, OR=0.740, 95%CI=[0.663, 0.827]) (Table 2).

**Table 2:** Genotype and allele distributions of rs2171513, rs1077667, and rs12609318

Parameter	Controls (n = 387)	AS patients (n = 497)	p-value	OR (95%CI)
rs2171513 C/T				
HWE	0.003	5.99678E-07		
Genotype				
CC	217(56.07%)	256	0.114	1.00
CT	130(33.59%)	167	NS	0.949(0.793- 1.136)
TT	40(10.34%)	74		0.694(0.490 - 0.983)
alleles				
C	564	679	**0.037**	1.00
T	210	315		0.856 (0.739 - 0.992)
rs1077667 G>A				
HWE	0.0006	4.00E-06		
Genotype				
GG	169(43.67%)	154	**0.000004**	1.00
GA	149(38.50%)	188		0.852 (0.733 - 0.992)
AA	69(17.83%)	155		0.578 (0.460 - 0.726)
alleles				
G	487	496	**4.59E-08**	1.00
A	287	498		0.740 (0.663 - 0.827)
rs12609318 A>G				
HWE	0.001	0.097		
Genotype				
AA	92(23.77%)	115	0.146	1.00
GA	228(58.91%)	267		1.019 (0.926 - 1.122)
GG	67(17.31%)	115		0.843 (0.674 - 1.054)
alleles				
A	412	497	0.178	1.00
G	362	497		0.935 (0.848 - 1.031)

p< 0.05 is considered of statistical significance.

### T allele of rs2171513 associated with susceptibility to AS

The dominant model of rs2171513 (CC + CT vs. TT) showed significant difference between patients and controls(*p* = 0.045, OR=1.053, 95%CI =[1.002, 1.107], power = 83%) and less T allele exists in patients (27.13% vs. 31.69%, *p* = 0.037, OR = 0.856, 95%CI=[0.739, 0.992]). However, no significant difference was found in the distribution of genotype (*p* = 0.114) or recessive model (*p* = 0.48) (Tables 2 and 6).

**Table 3:** BTLA rs2171513 genotypes and clinical indexes in AS patients

Parameters	rs2171513			p-value
	CC	CT	TT	
CRP	22.58(2.66, 23.70)	21.05(3.09, 24.60)	20.05(3.09, 23.30)	0.833
WBC	7.74 ± 2.09	7.61 ± 2.07	7.48 ± 1.55	0.722
C3	1.12 ± 0.27	1.09 ± 0.31	1.07 ± 0.27	0.813
C4	0.24 ± 0.08	0.26 ± 0.10	0.24 ± 0.09	0.814
RBC	4.88 ± 0.58	4.99 ± 0.64	4.91 ± 0.54	0.304
PLT	248 ± 94	257 ± 102	251 ± 82	0.525
Monocyte	0.44 ± 0.17	0.42 ± 0.15	0.43 ± 0.14	0.485
Lymphocyte	2.06 ± 0.62	1.93 ± 0.59	1.98 ± 0.59	0.070
Neutrophil	5.03 ± 1.85	4.97 ± 1.87	4.82 ± 1.25	0.863

The data are presented as mean ± SD.

The data of CRP are presented as mean (lower quartile, upper quartile).

p< 0.05 is considered of statistical significance.

**Table 4:** BTLA rs1077667 genotypes and clinical indexes in AS patients

Parameters	rs1077667			p-value
	GG	GA	AA	
CRP	23.17(2.79, 24.10)	20.63(2.60, 26.43)	17.67(2.54, 18.7)	0.570
WBC	7.80 ± 2.15	7.45 ± 1.98	7.76 ± 1.86	0.2
C3	1.10 ± 0.31	1.15 ± 0.29	1.05 ± 0.24	0.506
C4	0.23 ± 0.08	0.27 ± 0.10	0.24 ± 0.09	0.185
RBC	4.98 ± 0.59	4.88 ± 0.64	4.90 ± 0.54	0.135
PLT	250 ± 91	255 ± 99	250 ± 95	0.902
Monocyte	0.43 ± 0.15	0.42 ± 0.16	0.45 ± 0.16	0.334
Lymphocyte	2.07 ± 0.64	1.98 ± 0.63	1.96 ± 0.55	0.431
Neutrophil	5.06 ± 1.88	4.86 ± 1.72	5.03 ± 1.71	0.652

The data are presented as mean ± SD.

p< 0.05 is considered of statistical significance.

**Table 5:** BTLA rs12609318 genotypes and clinical indexes in AS patients

Parameters	rs12609318			p-value
	AA	GA	GG	
CRP	20.77(5.25, 17.20)	21.58(2.72, 25.40)	18.47(2.49, 22.00)	0.553
WBC	7.79 ± 2.23	7.51 ± 1.91	7.85 ± 1.94	0.273
C3	1.08 ± 0.29	1.14 ± 0.30	1.04 ± 0.25	0.507
C4	0.25 ± 0.09	0.25 ± 0.10	0.23 ± 0.07	0.728
RBC	4.88 ± 0.60	4.92 ± 0.62	4.95 ± 0.54	0.864
PLT	258 ± 93	256 ± 100	236 ± 82	0.205
Monocyte	0.44 ± 0.17	0.43 ± 0.15	0.44 ± 0.17	0.807
Lymphocyte	2.01 ± 0.59	2.01 ± 0.61	1.99 ± 0.63	0.965
Neutrophil	5.16 ± 1.96	4.86 ± 1.67	5.07 ± 1.79	0.460

The data are presented as mean ± SD.

p< 0.05 is considered of statistical significance.

**Table 6:** Association between rs2171513 C/T, rs1077667 G>A and rs12609318 A>G genotypes

SNP	Controls	Patients	p-value	OR (95%CI)
rs2171513				
CC+CT	347	423	**0.045**	1.053 (1.002 - 1.107)
TT	40	74		
rs1077667				
GG+GA	318	342	**0.000006**	1.194 (1.108 - 1.287)
AA	69	155		
rs12609318				
AA+GA	320	382	**0.034**	1.076 (1.007 - 1.150)
GG	67	115		
Recessive				
rs2171513				
TT+CT	170	241	0.48	0.948(0.817 - 1.100)
CC	217	256		
rs1077667				
AA+GA	218	343	**0.000102**	0.816 (0.734 - 0.907)
GG	169	154		
rs12609318				
GG+GA	295	382	0.825	0.992 (0.921 - 1.068)
AA	92	115		

p< 0.05 is considered of statistical significance.

### The GG genotype of rs12609318 relates with the onset of AS

The dominant model of rs12609318 showed significant difference between patients and controls(*p* = 0.034, OR = 1.076, 95%CI [1.007, 1.150], power = 48%), though neither genotype nor allele showed statistically significant difference (*p* = 0.146 and 0.178, respectively)(Table 2, 6).

### Clinical indexes of AS patients

AS patients were divided into three groups according to the three genotypes of each SNP. All the clinical indexes including C-reactive protein (CRP), white blood cell (WBC), red blood cell (RBC), platelet (PLT), monocyte, lymphocyte, neutrophil, complement 3 and 4 (C3 and C4) levels data did not show any significant differences among three genotypes (all *p*> 0.05) (Tables 3-5).

## DISCUSSION

In this case-control study of AS among adults from a Chinese Han population, we indicated that the associations between those four SNPs and the susceptibility to AS. We found the frequency of the AA genotype of rs1077667 in LIGHT gene is higher in AS patients than in controls (p = 4.00E-06), and the frequency of A allele is also higher in the patients (37.1% vs. 50.1%, p =4.59E-08). Meanwhile, there was a correlation between the TT genotype of rs2171513 in BTLA gene and the susceptibility to AS in the dominant model (*p* = 0.045), and the T allele is of a higher ratio in the patients (*p* = 0.037). The dominant model of rs12609318 in LIGHT gene was also found a statistical significant difference (*p* = 0.034). The frequency data of our control group matches up with the data shown in HapMap though they are both not in HWE (p < 0.05) (Figures 1-3).

AS is a complicated polygenic disease correlating with multiple genetic mutations. And here in this study, we found rs1077667 associated with the susceptibility to AS. Significant difference was found in the distribution of genotypes of rs1077667 (p = 4.00E-06), which indicated that people with AA genotype of rs1077667 had more probability to get AS. As for alleles (p = 4.59E-08), we can do a reasonable speculation that A allele is highly likely to participate in the pathological process of AS. The dominant model and recessive model both showed statistically significant difference between patients and controls (p = 6.00E-06 and 0.000102, respectively), which supported our speculation.

LIGHT has two different forms, membrane-bound form and soluble form. Although the mechanism of transformation between the two different forms is still not clear, membrane-bound LIGHT is showed to play an important role in the survival of CD4+ memory T-cell and act as a co-stimulatory molecule, but not as strong as CD28 [13]. Besides, previous studies showed that level of serum-soluble LIGHT is always higher in patients than in controls. The association between rs1077667 and LIGHT level is pointed out that rs1077667 AA carriers have the highest LIGHT level, Thus we got the conclusion that the mutation from G allele to A allele leads to over expression of LIGHT and activated the inflammatory process which leads to the development of AS [7]. And the mutation of rs12609318 may act the similar way.

As for BTLA, T allele of rs2171513 appears to be more in the AS patients than controls (p = 0.037, OR = 0.856, 95%CI = [0.739, 0.992]), that may due to the mutant related to the repression of HVEM-BTLA complex, which has been shown to decrease T cell activity *in vivo* and limit homeostatic expansion of CD4^+^ and CD8^+^ T cells [14, 15]. The dominant model (AA + GA vs. GG)of rs12609318 shows statistical significance (p = 0.034, OR = 1.076, 95%CI = [1.007, 1.150]),which indicated that the AA and GA genotypes are associated with the hazard of AS.

In this study, we found three SNPs rs2171513, rs1077667 and rs12609318 correlated with the susceptibility to AS. But how those mutations of SNPs acted in the transcription process remained unknown and our laboratory did not have the access to this part of research. And more research for further exploration of the molecular mechanism should be done. As for rs2171513 and rs12609318, although their genotypes and alleles did not show statistical significance in our study, it might due to the limitation by the shortage of sample size.

In conclusion, in this study we found SNPs rs2171513, rs1077667 and rs12609318 are associated with the susceptibility to AS. But further studies with a larger sample size are necessary to enhance the power. Moreover, the explanation of how exactly the expression level was influenced need more studies and experiments. Many a little makes a mickle, each step we head forward will give us a better view of AS.

## MATERIALS AND METHODS

### Study subjects

This study was approved by the Institutional Review Board of Sichuan University. All participants provided their written informed consent to participate in this study. A total of 497 AS patients were diagnosed by rheumatologists according to New York clinical criteria in West China Hospital of Sichuan University [16]. And 387 gender-, age-, and ethnicity-matched healthy controls were enrolled in this study during Jan. 1 2014 to Nov. 1 2014. C-reactive protein (CRP), white blood cell (WBC), red blood cell (RBC), platelet (PLT), monocyte, lymphocyte, neutrophil, complement 3 and 4 (C3 and C4) levels were collected when patients were recruited. All study subjects were of Han ethnicity and lived in the west of China.

### Genomic DNA extraction

Blood samples (3 mL) were collected in EDTA-coated tubes, and genomic DNA was isolated from whole blood samples using the whole blood DNA kit (Roche Diagnostics; Penzberg, Bavaria, Germany). DNA was extracted from 200μL of the whole blood, according to the manufacturer’s protocol. The extracted DNA was assessed for purity, yield, and concentration on a spectrophotometer (Bio-RAD; Hercules, CA, USA). Purity was monitored by the A260/A280 ratio. DNA was diluted to 10 ng/μL for working solutions, and isolated DNA was stored at -20°C. The details of methods were illustrated in the former study [17].

### Polymerase chain reaction and high-resolution melting method

The rs2171513 C>T in BTLA gene,rs1077667 G>A and rs12609318 A>G in the LIGHT gene were assessed. The SNPs we selected are those haven’t been studied, and more than 5% people are of wild type to be more representative. Polymerase chain reaction (PCR) and melting curve analyses were performed under the same conditions in a 96-well plate on the Light Cycler 480 (Roche Diagnostics). The primers were designed to a small fragment surrounding the polymorphisms and avoided the presence of other sequence variations in the primer region. The rs2171513 C/T PCR primers were 5’-CCGAACTCAAGACTGGCAAGA-3’ (forward) and 5’-GACCCAAGCACTAACATGAACATT -3’ (reverse). The rs1077667 G>A PCR primers were 5’-ATGCCCAACACCATACCCCT-3’ (forward) and 5’-AGACAAAGACAGACAACGTGAATAC-3’ (reverse). The rs12609318 A>G PCR primers were 5’-TCCTTCCTTTCAATCAGCAAGC-3’ (forward) and 5’- ACAGCTTTGTAATGTTTGCCAATT -3’ (reverse). Reaction mixtures contained 1.0 μL purified genomic DNA (10 ng/μL), 0.5 μL forward primer, 0.5 μL reverse primer, 1.4 μL 20×EVA-GREEN, 0.5 μLdNTP (10 mM), 0.2 μL Hot Star Taq® Plus DNA polymerase, 2 μL 10× buffer, and 1 μL 50 mM MgCl2. Real-time PCR was performed with the following conditions: an initial denaturation step at 95°C for 15 min, continued with 50 cycles of 95°C for 10 s, 60°C for 15 s, and 72°C for 20 s. After the amplification phase, a melting curve analysis was performed at 95°C for 1 min, 40°C for 1 min, 65°C for 1 s, and finally slow heating at 0.01°C/s to 95°C.

### HRM analysis

Collected data were analyzed by the Light Cycler 480 Gene Scanning software v1.2 (Roche Diagnostics). Software programs used the following analysis method: normalization by selecting linear regions before and after the melting transition, temperature shifting by selecting the threshold, then automatic grouping by calculation. The exact same setting of the normalization was used for all experiments. And all methods were performed in accordance with the relevant guidelines and regulations.

### Statistical analysis

The Hardy-Weinberg equilibrium (HWE) for rs2171513 C>T, rs1077667 G>A and rs12609318 A>G were tested by the χ^2^ test. The difference of the frequency distribution was analyzed by the χ^2^ test. A difference of p < 0.05 was considered statistically significant. The difference in data for clinical features and pathology grading among different genotypes was analyzed using the Kruskal–Wallis tests. All analyses above were performed with SPSS software (version 19.0, SPSS; Chicago, IL, USA). Statistical power analysis was done with Quanto software (version 1.2.4; University of Southern California; Los Angeles, CA, USA) to determine the power to detect the association of rs2171513 C>T, rs1077667 G>A and rs12609318 A>G alleles with susceptibility to AS at the designated significance level.
